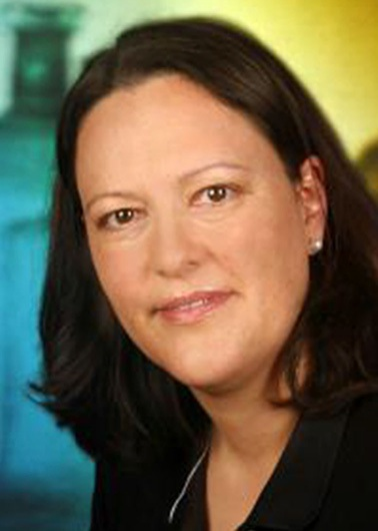# Non‐Oxidative Enzymatic (De)Carboxylation of (Hetero)Aromatics and Acrylic Acid Derivatives

**DOI:** 10.1002/adsc.201900275

**Published:** 2019-05-17

**Authors:** Stefan E. Payer, Kurt Faber, Silvia M. Glueck

**Affiliations:** ^1^ Institute of Chemistry University of Graz Heinrichstrasse 28 8010 Graz Austria

**Keywords:** biocatalysis, carbon dioxide, CO_2_ fixation, decarboxylases, (de)carboxylation, reaction mechanisms, substrate scope

## Abstract

The utilization of carbon dioxide as a C_1_‐building block for the production of valuable chemicals has recently attracted much interest. Whereas chemical CO_2_ fixation is dominated by C−O and C−N bond forming reactions, the development of novel concepts for the carboxylation of C‐nucleophiles, which leads to the formation of carboxylic acids, is highly desired. Beside transition metal catalysis, biocatalysis has emerged as an attractive method for the highly regioselective (de)carboxylation of electron‐rich (hetero)aromatics, which has been recently further expanded to include conjugated α,β‐unsaturated (acrylic) acid derivatives. Depending on the type of substrate, different classes of enzymes have been explored for (i) the *ortho*‐carboxylation of phenols catalyzed by metal‐dependent *ortho*‐benzoic acid decarboxylases and (ii) the side‐chain carboxylation of *para*‐hydroxystyrenes mediated by metal‐independent phenolic acid decarboxylases. Just recently, the portfolio of bio‐carboxylation reactions was complemented by (iii) the *para*‐carboxylation of phenols and the decarboxylation of electron‐rich heterocyclic and acrylic acid derivatives mediated by prenylated FMN‐dependent decarboxylases, which is the main focus of this review. Bio(de)carboxylation processes proceed under physiological reaction conditions employing bicarbonate or (pressurized) CO_2_ when running in the energetically uphill carboxylation direction. Aiming to facilitate the application of these enzymes in preparative‐scale biotransformations, their catalytic mechanism and substrate scope are analyzed in this review.

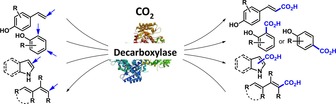

## 1 Introduction

Although carbon dioxide (CO_2_) is predominantly regarded as an undesired greenhouse gas, its utilization in carboxylation reactions as a C_1_‐building block for the synthesis of value‐added compounds has become highly attractive in recent years. Being a weak electrophile, carbon dioxide can react with N‐, O‐, and C‐nucleophiles in a formal addition reaction, leading to carbamates, ureas, carbonate esters^1^ and carboxylic acids.^2^ The fact that these reactions proceed with 100% atom economy and the low (no) cost of the reagent renders CO_2_ an attractive resource for industrial processes. In particular, the production of urea (∼95 Mt/a), organic (poly)carbonates (∼150 kt/a) and salicylic acid (∼70 kt/a) proceeds on a very large scale, in addition to the (chemical) reduction of CO_2_ to (predominantly) methanol (∼60 Mt/a). Despite these impressive large‐scale processes, the contribution of ‘chemical’ CO_2_ fixation during the synthesis of organic molecules is only marginal (≤1%, ∼200 Mt/a) compared to its massive generation in combustion processes (∼37 Gt/a)^3^ for the generation of energy, which proceeds with a modest efficiency of 30–35%, dictated by the second law of thermodynamics.^4^ Consequently, carboxylation for the synthesis of organic compounds is irrelevant in the balance of the carbon cycle.^5^ In contrast to industrial processes, carboxylation is only scarcely used in small molecule synthesis, owing to the low reactivity and kinetic and thermodynamic inertness of CO_2_, which requires significant catalytic activation and/or substantial energy input. The harsh reaction conditions required in traditional carboxylation processes^6^ are often the cause for limited selectivities. Over the last decades, various strategies have been developed in order to overcome these limitations. Traditionally, beside strong nucleophilic organometallic (organolithium or Grignard) reagents,^7^ which often suffer from poor chemoselectivity, highly strained substrates (such as epoxides, aziridines, etc.), which are restricted to C−O and C−N bond‐forming reactions, were employed to overcome the energetic barrier to yield the corresponding linear or cyclic (poly)carbonates/carbamates.^2^ The enormous progress in transition metal catalysis and cross‐coupling reactions has paved the way for the most desired carbon dioxide fixation *via* C−C bond formation to furnish the corresponding carboxylic acids.^2^ The latter is the second most abundant functional group occurring in small molecules produced by chemical synthesis.^8^ The most commonly used pre‐activated substrates for transition metal‐catalyzed carboxylations include allylstannanes, organoboronic esters, organozinc reagents and aryl halides.^9^ Unsaturated compounds (olefins, allenes, alkynes) can be reductively carboxylated in a formal ‘hydrocarboxylation’^10^ at the expense of a co‐reactant providing hydride species.2d, 11 The progress in organocatalytic^12^ and electrochemical^13^ methods further expands the scope of carbon dioxide fixation reactions in organic synthesis. In this context, modern reaction/process engineering tools (such as continuous flow technologies,^14^ etc.) are being developed for process design.

In nature, four major pathways for biological CO_2_ fixation have been evolved:^8^, ^15^ (i) the Calvin–Benson–Bassham cycle, (ii) the Arnon–Buchanan (‘reductive TCA’) cycle, (iii) the Wood–Ljungdahl (‘reductive AcetylCoA’) cycle, and the (iv) acetyl‐CoA pathways. Common to all these pathways is the fact that they belong to the primary metabolism, hence the enzymes involved are highly specialized (evolved) for one (or a few) substrate(s).^16^ Consequently, they are generally of limited use for the biotransformation of non‐natural organic compounds. In contrast, enzymes involved in defence and detoxification – the secondary metabolism – are ‘generalists’, as they act on a broad variety of substrates and hence are more useful as biocatalysts for organic synthesis.^8^, ^17^, ^18^

The major goal of detoxification is making (lipophilic) toxins more polar to assist their removal from the cell. Among several pathways (such as oxidation, glycosylation, phosphorylation, sulfation, peptide conjugation), carboxylation is a viable option to convert lipophilic aromatics into water‐soluble carboxylic acids.

Arenes are widely distributed in nature and serve as substrates for aerobic and anaerobic organisms. Most natural aromatic compounds are derived from secondary plant metabolism and often contain phenolic groups, such as products from lignin degradation, tannins and flavonoids, which predominantly consist of substituted phenols, benzaldehydes, benzoic and cinnamic acids. Whereas oxidative biodegradation of aromatics mainly involves oxygenases, anaerobic bacteria apply reductive pathways^19^ or redox‐neutral carboxylation.^20^

The utilization of nature's tools – (de)carboxylases – to establish biocatalytic concepts for the (de)carboxylation of (hetero)aromatic substrates and conjugated α,β‐unsaturated carboxylic (acrylic) acids as a sustainable alternative to chemical methods (such as the Kolbe–Schmitt process) has been intensively investigated over the past years. In this review, various methods are described together with the mechanism of the respective enzymes and their substrate tolerance with particular focus on the very recently explored reversible (de)carboxylation reactions mediated by prenylated FMN‐dependent decarboxylases. The latter are applicable to the decarboxylation of acrylic acid derivatives as well as the *para*‐carboxylation of phenols. Furthermore, a general overview is given for the *ortho*‐ and side‐chain (de)carboxylation of phenol‐ and hydroxystyrene‐type substrates by metal‐dependent and cofactor‐independent decarboxylases, both summarized in a recent comprehensive review by I. C. Tommasi.^21^

## 2 Enzymatic (De)Carboxylation

**Scheme 1:**
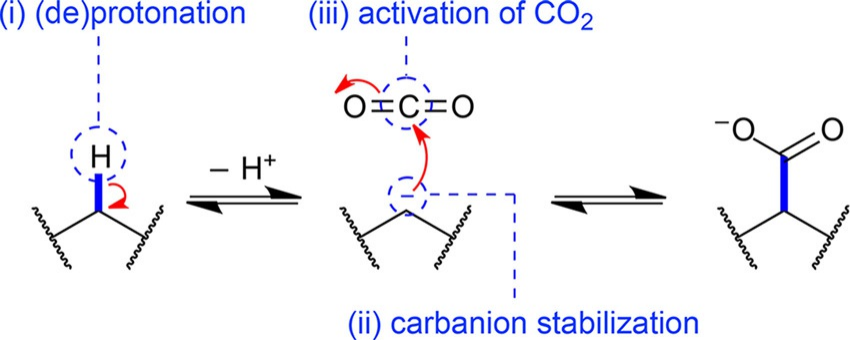
Generic carboxylation of a C−H bond: deprotonation forms a carbanion, which attacks a CO_2_ electrophile.

Enzymes have developed a diverse set of strategies for the attachment or release of CO_2_ to or from various substrates by exploiting metal ions or cofactors, like pyridoxal phosphate (PLP), thiamine diphosphate (ThDP, vitamin B6) or prenylated FMN (prFMN).^22^ The following requirements to facilitate the carboxylation of a C−H bond to install a carboxylate group need to be fulfilled (Scheme 1): (i) abstraction of a proton (commonly with a p*K*_a_ of 15–18) to generate an intermediary carbanion equivalent, (ii) stabilization of the accumulating negative charge through delocalization within the substrate structure (mostly as metal ion‐chelated enolate) or a cofactor conjugate, and (iii) activation of carbon dioxide toward nucleophilic attack of the carbanion equivalent.^23^

The question of whether CO_2_ or bicarbonate serves as a uniform co‐substrate in enzymatic carboxylation is still under debate.^24^ Although the majority of carboxylases utilize the electrophilic, yet poorly water‐soluble carbon dioxide, fewer enzymes like phosphorenol pyruvate carboxylase and biotin‐dependent carboxylases exploit the considerably less reactive (but water‐soluble) bicarbonate ion for carboxylation.^23^ These enzymes are proposed to possess a dual carboxylase/carbonic anhydrase activity, which allows them to interconvert bicarbonate and CO_2_ and use the latter as actual co‐substrate for carboxylation. On the other hand, a specific motif responsible for binding CO_2_ in carboxylases in analogy to the CO_2_ binding site of phosphoenol pyruvate carboxykinase,^25^ has not been identified yet.

## 3 Biocatalytic (De)Carboxylation of (Hetero)Aromatics and α,β‐Unsaturated (Acrylic) Acids

**Figure 1:**
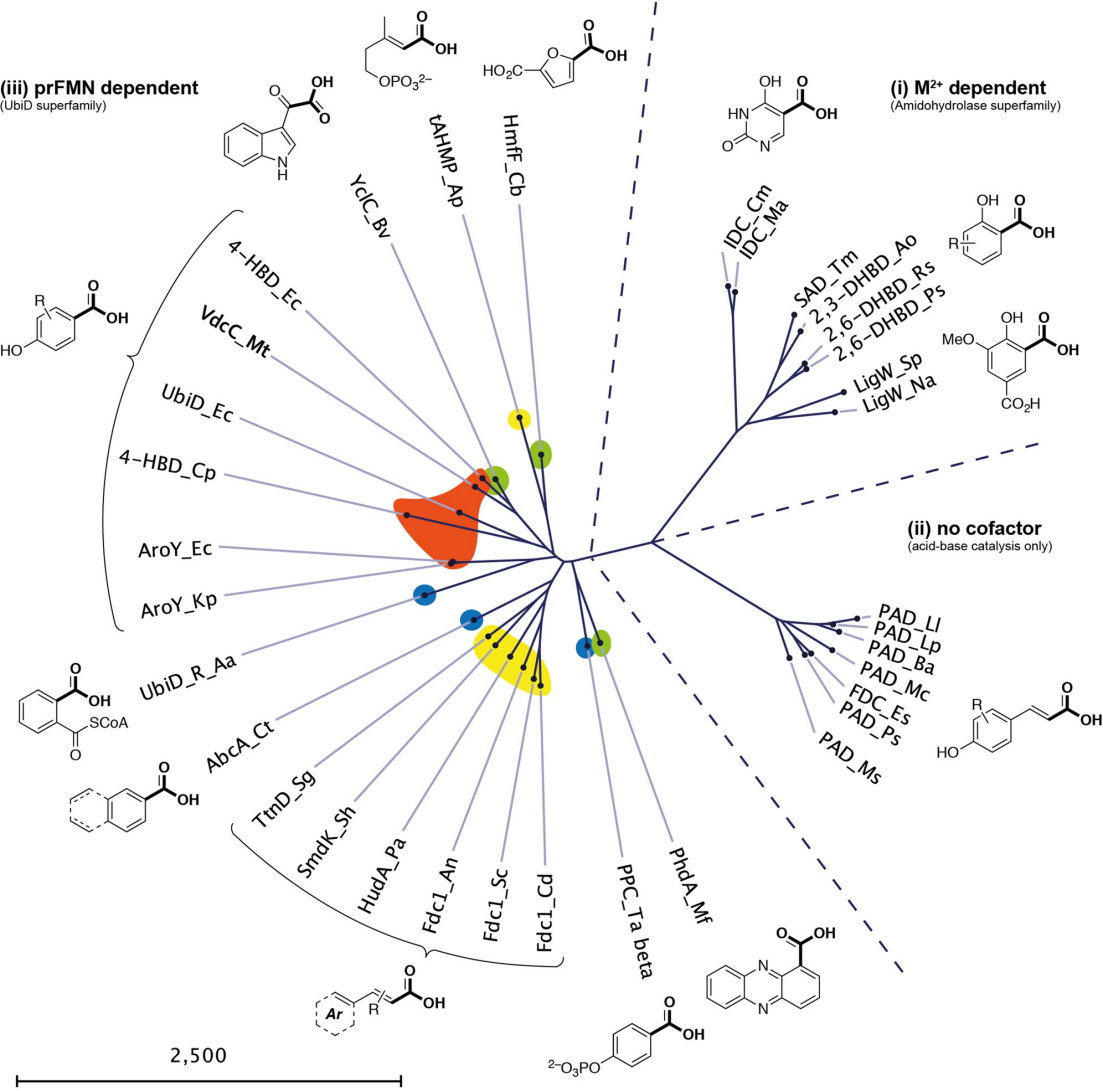
Phylogenetic tree showing an overview of the three major classes of biocatalytically relevant arene (de)carboxylases.

Compared to carboxylases and decarboxylases (carboxylyases, EC 4.1.1.X) acting on amino acids, carbohydrates (including oxalate and pyruvate) and other aliphatic substrates, which are widespread in primary metabolic pathways, less enzymes are known to catalyze the (de)carboxylation of aromatic substrates, found in secondary metabolism. Among them, a remarkable number of enzymes for potential biocatalytic applications has been identified which can be classified into three major categories based on mechanistic aspects (Figure 1): (i) divalent metal‐dependent decarboxylases from the amidohydrolase superfamily, (ii) cofactor‐ and metal‐independent phenolic acid decarboxylases, and (iii) prenylated FMN‐dependent (prFMN) decarboxylases from the UbiD superfamily. The latter display activity for a wide array of structurally diverse substrates [Figure 1, (iii)] and are associated with the reversible (de)carboxylation of phenols (highlighted in orange), heteroarenes (green), α,β‐unsaturated (acrylic) acids (yellow), and other arenes (blue), respectively. Since prFMN was discovered only recently the biocatalytic characterization of prFMN‐dependent decarboxylases is at its early stage assuming that the identification of further enzyme candidates is most likely and their use for biocatalytic application is an intriguing future aim. Several 3,4‐26 and 4,5‐dihydroxyphthalate^27^ decarboxylases have been identified, however, it is not clear yet whether these enzymes are members from the UbiD superfamily.

Owing to its stability and straightforward reconstitution with the prFMN cofactor, the Fdc subfamily can be regarded as the most versatile and applicable decarboxylation biocatalyst of the UbiD family as of now.

In order to drive the equilibrium towards the energetically disfavored carboxylation direction, an excess of bicarbonate (usually 2–3 M)^21^ is most commonly applied as CO_2_ source, alternatively pressurized (∼30 bar)^28^ or sub‐/supercritical^29^ carbon dioxide is used to a minor extent.

### 3.1 Divalent Metal‐Dependent Decarboxylases

All of the metal‐dependent decarboxylases [Figure 1, (i)] identified so far are members of the amidohydrolase superfamily (AHS). They share significant structural and mechanistic similarities, in particular the characteristic (β/α)_8_‐barrel fold harbouring one catalytically relevant divalent metal ion in the active site.^30^ Whereas the overall sequence similarity between distinct subclasses is rather low (around 30%),^31^, ^32^ several amino acid residues relevant for catalysis are conserved. Although members of the AHS commonly catalyze the hydrolysis of ester and amide bonds attached to either a carbon or phosphorus atom on a wide range of structurally diverse substrates,^30^ some members obviously evolved to catalyze the reversible decarboxylation of benzoic acid derivatives and nitrogen‐heterocyclic derivatives thereof.

**Scheme 2:**
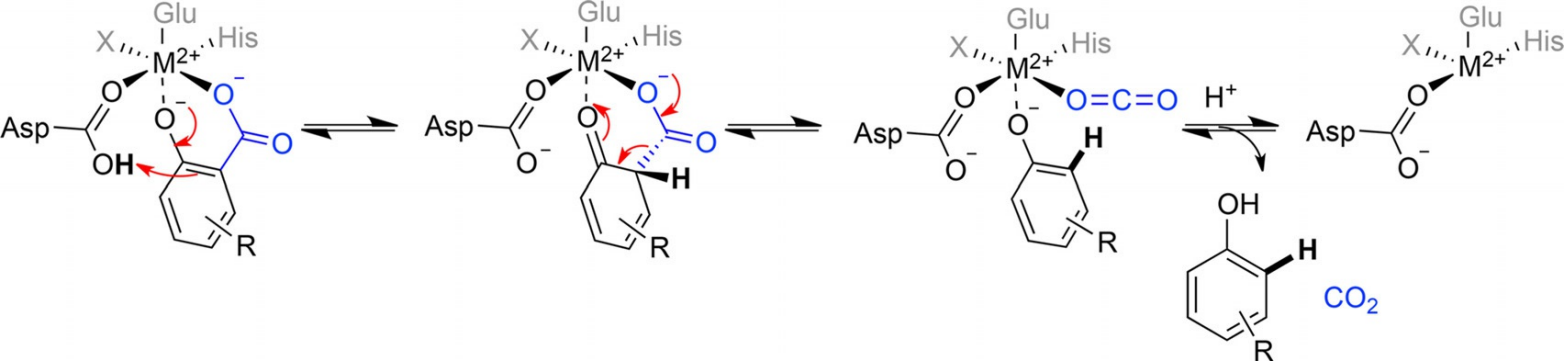
General mechanism for the *ortho*‐(de)carboxylation of M^2+^‐dependent decarboxylases from the amidohydrolase superfamily; M^2+^=Zn^2+^, Mn^2+^.

Density functional theory (DFT) calculations employing large active site models based on crystal structures of *ortho*‐benzoic acid decarboxylases (*o*‐BDCs) strongly support a general mechanistic proposal, which resembles a (reverse) electrophilic aromatic substitution by feasible energy barriers and bears a strong resemblance to the Kolbe–Schmitt reaction.^31^, ^32^ In more detail, this general mechanism involves the metal ion (predominantly manganese or zinc) chelating the carboxylate and phenolate group of the arene substrate, thereby stabilizing their negative charge. Coordination arranges the nucleophilic phenolate for protonation at the carboxylate *ipso*‐carbon by a nearby conserved catalytic acid (Asp), which is accompanied by dearomatization of the arene. The latter is restored upon loss of carbon dioxide, which dissociates together with the decarboxylated phenol from the metal center (Scheme 2).^31^, ^32^, ^33^

**Figure 2:**
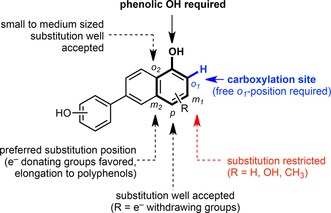
General substrate model of M^2+^‐dependent decarboxylases: *ortho*‐benzoic acid decarboxylases (*o*‐BDCs) and 5‐carboxyvanillate decarboxylases (LigWs) for the reversible (de)carboxylation of phenol‐type substrates.

The stability and broad substrate tolerance of *o*‐BDCs^8^, ^21^ (such as 2,3‐dihydroxybenzoic acid decarboxylases from *Aspergillus* and *Fusarium* species, salicylic acid decarboxylase from *Trichosporon moniliiforme*, and 2,6‐dihydroxybenzoic acid/γ‐resorcylate decarboxylases from *Agrobacterium*, *Rhizobium*, *Pandoraea*, *Rhodococcus* and *Polaromonas* species)29a, 34 recommended them as excellent biocatalysts for the regioselective *ortho*‐carboxylation of phenols also on a preparative scale. This also applies for 5‐carboxyvanillate decarboxylases (LigWs, from *Sphingomonas* and *Novosphingobium* species), although they exhibit a more restricted substrate tolerance.^21^, ^35^ The minimal structural requirements (shown in Figure 2) are characterized by a phenolic motif, in which the aromatic system supports the resonance stabilization of the carbanion intermediate. The phenolic OH group seems to be mandatory, since NH_2_ (aniline) and SH variants (thiophenol) are not accepted due to inaccurate electronic (lower or significantly higher p*K_a_* of the SH or NH protons, respectively) and/or structural (atomic diameter) properties. Carboxylation is inevitably associated to a free *ortho*‐position.^21^, 33a Regarding the substitution pattern, the *meta*‐position (*m_2_*, Figure 2) opposite to the carboxylation site is most flexible tolerating weakly e^−^‐withdrawing (halogens) and in particular e^−^‐donating groups (alkyl, alkoxy, hydroxy, and amino functionalities), which can be even extended to (conjugated) aromatic systems to encompass remarkably large polyphenols, such as resveratrol.33a, 34b The *meta*‐position (*m_1_*) adjacent to the carboxylation site is less flexible, only OH and CH_3_ substitution is reported. The *para*‐position favours weak e^−^‐donors and ‐acceptors and seems to be unique in the acceptance of strongly e^−^‐withdrawing carbonyl/carboxyl moieties as well as elongated (un)saturated propionic/acrylic acids.^35^ Substituents in the non‐reactive *ortho*‐position (*o_2_*) are well accepted as long as they are small. The tolerance of multiple functionalized substrates is diverse and depends on the electronic and steric nature of the substituents. In general, the electronic properties of the functionalization seem to play a more significant role than steric effects.

In contrast, the biocatalytic characterization of another member of this class of enzymes – *iso*‐orotate decarboxylase (IDCase) – acting on a heterocyclic substrate analogue, revealed a very narrow substrate specificity and only acts in the energetically favoured decarboxylation direction.^31^

Process engineering, such as the addition of quaternary ammonium salts to induce the precipitation of the corresponding carboxylated products as ion pairs^36^ as well as enzyme engineering, e.g., for *para*‐aminosalicylic acid production34d significantly improved the efficiency and operability of biocatalytic *ortho*‐carboxylations, which is particularly important for their industrial application.

### 3.2 Cofactor‐Independent Decarboxylases

Cofactor‐independent phenolic acid decarboxylases [PADs, Figure 1, (ii)] are involved in the biodegradation of coumaric acid derivatives^37^ (arising from the breakdown of lignin) and have been explored to catalyze the reversible carboxylation of styrene‐type substrates at the expense of bicarbonate as carboxylating agent.^38^ Several enzymes originating from bacteria or plants have been biocatalytically characterized: phenolic acid decarboxylase from *Lactobacillus plantarum*, *Bacillus amyloliquefaciens, B. licheniformis, B. subtilis, Mycobacterium colombiense, Methylobacterium* sp., *Pantoea* sp., *Candida guilliermondii, Pediococcus pentosaceus, Conocephalum japonicum* as well as ferulic acid decarboxylase from *Enterobacter* sp. and *B. pumilus*.^38^, ^39^

PADs exclusively act on the β‐carbon of the styrene side chain to yield the corresponding (*E*)‐coumaric acids, which is of particular interest since apart from the Pd‐catalyzed alkenyl bond carboxylation of functionalized 2‐hydroxystyrenes yielding coumarin derivatives,^40^ general chemical methods are lacking.

**Scheme 3:**
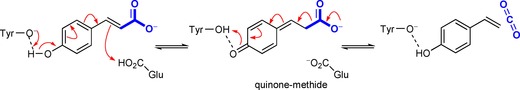
General catalytic acid–base mechanism for the side chain (de)carboxylation by cofactor‐independent PADs.

PADs strictly require a fully conjugated system between the C_β_ carbon atom and the mandatory *para*‐hydroxy group to facilitate a substrate‐based resonance stabilization of the negative charge *via* a quinone methide intermediate in the acid–base‐catalyzed (de)carboxylation reaction (Scheme 3).^41^, ^42^

**Figure 3:**
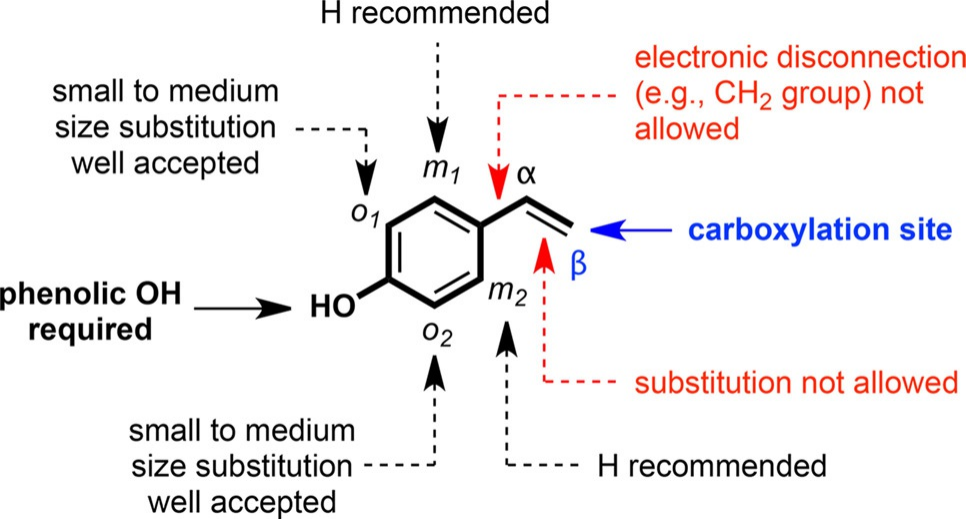
General substrate model of phenolic acid decarboxylases catalyzing the reversible (de)carboxylation of hydroxystyrene type substrates.

The overall robustness and substrate tolerance of PADs is more limited compared to those of *o*‐BDCs.^21^, ^43^ The introduction of functionalization is mainly restricted to the *ortho*‐positions (*o_1_* and/or *o_2_*) tolerating e^−^‐donating (alkyl, alkoxy) and ‐withdrawing groups (halogens), whereas *meta*‐substitution (*m_1_*, *m_2_*) led to unstable products. Variations in the α‐ or β‐position of the styrene side chain as well as replacement of the *para*‐OH group (e.g., by Cl, OMe, NH_2_) is prohibited and causes a total loss of enzymatic activity. In general, the steric properties of the substituents seem to play a more crucial role than electronic effects (Figure 3).

### 3.3 prFMN‐Dependent Decarboxylases

The remarkably broad substrate tolerance, excellent regioselectivity, and the robustness of most *ortho*‐benzoic acid decarboxylases facilitate their widespread utilization in biocatalytic applications for the *ortho*‐specific (de)carboxylation of phenolic substrates towards the corresponding aromatic carboxylic acids as a biocatalytic equivalent to the Kolbe–Schmitt^44^ reaction. An enzymatic route to the regiocomplementary *para*‐carboxylation of phenols is highly desired since the chemical counterpart suffers from unsatisfactory regioselectivities.

**Scheme 4:**
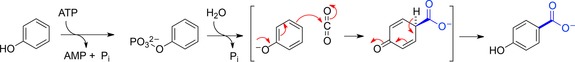
*para*‐Carboxylation of phenol *via* phenyl phosphate catalyzed by phenyl phosphate carboxylase. Hydrolysis of the phenyl phosphate generates a reactive phenolate anion, which attacks a CO_2_ electrophile in the active site of the enzyme following an S_E_Ar mechanism.

*para*‐Selective CO_2_ fixation was described for phenylphosphate carboxylases (PPC), which require ATP‐dependent activation of the phenol substrate *via* phosphorylation prior to the carboxylation step (Scheme 4).^45^ Two enzymes from *Pseudomonas* strain K172 (PsPPC)45a, 46 and *Thauera aromatica* (TaPPC, Mn^2+^ dependent)45d, 47 have been purified and characterized. Although the biocatalytic applicability of TaPPC with a TON of up to 16000 was demonstrated after stabilization of the oxygen‐sensitive enzymes by immobilization on Agar beads,45d the scope of this enzyme class is limited to phenyl phosphate and catechyl phosphate^48^ substrates. Both the narrow substrate specificity and the dependence on expensive ATP limit the usability of these enzymes for biocatalytic applications.

**Scheme 5:**
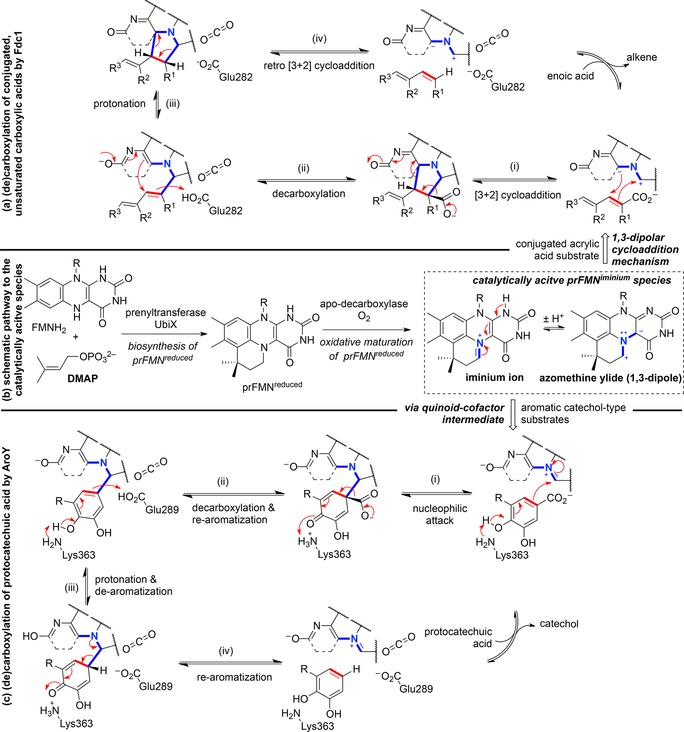
(a) prFMN‐dependent catalytic 1,3‐dipolar cycloaddition mechanism for the (de)carboxylation of conjugated, unsaturated substrates by ferulic acid decarboxylase (Fdc1) from *Aspergillus niger*; (b) simplified schematic representation of the production of prFMN^iminium^; (c) prFMN‐dependent catalytic mechanism for the (de)carboxylation of catechol‐type substrates *via* a quinoid‐cofactor intermediate by 3,4‐dihydroxybenzoic acid decarboxylase (AroY) from *Enterobacter cloacae*.

Just recently, an alternative to the ATP‐dependent *para*‐carboxylation was discovered. With the identification and subsequent characterization of a novel cofactor, first described as ‘modified FMN’ by Marsh et al.^49^ and later identified as prenylated flavin mononucleotide (prFMN) by Leys et al.,^50^ new concepts for biocatalytic (de)carboxylation strategies emerged. As a consequence of these seminal studies, numerous decarboxylases of the UbiD superfamily [named after UbiD involved in the ubiquinone (co‐enzyme Q) biosynthesis pathway in prokaryotes^51^] depending on this modified flavin cofactor were identified and partially characterized.22a Biosynthetically, the prFMN cofactor is provided by an associated prenyltransferase (UbiX) under anaerobic conditions. This enzyme uses the unusual C_5_‐metabolite γ,γ‐dimethylallyl monophosphate (DMAP) as co‐substrate to build up the prenyl moiety resulting in a fourth six‐membered ring between N‐5 and C‐6 of the isoalloxazine ring system of the reduced flavin [Scheme 5, (b)]. In contrast, the fungal UbiX analogue Pad1 uses the (metabolically more common) diphosphate DMAPP instead of DMAP for producing prFMN.^52^ In order to obtain the catalytically active iminium species of the cofactor (prFMN^iminium^), oxidative maturation of reduced prFMN (prFMN^reduced^) by molecular oxygen in the presence of *apo*‐decarboxylase appears to be crucial [Scheme 5, (b)].^53^ Although the exact oxidation mechanism of the reduced UbiX product to prFMN^iminium^ is unknown as of yet, a Glu‐Arg‐Glu motif as catalytic acid in the active site of the decarboxylase seems to assist this process.^54^ Notably, the prenyl modification in its catalytically active iminium form entirely eliminates the chemical properties of FMN as a redox mediator for hydride transfer and confers a 1,3‐dipolar azomethine ylide and electrophilic iminium ion character, respectively [Scheme 5, (b)].^55^

#### 3.3.1 Mechanistic Aspects of prFMN‐Dependent Decarboxylases

Detailed mechanistic scenarios were reported for the prFMN‐assisted (de)carboxylation of two structurally distinct (aromatic) substrates regarding the position of the carboxylic acid, either directly attached to the aromatic system^56^ or linked *via* a C=C bridge.22a, 50

Fdc1 (ferulic acid decarboxylase) from *Aspergillus niger* catalyzes the reversible decarboxylation of α,β‐unsaturated carboxylic acids employing the azomethine ylide form of a catalytically active prFMN iminium species [Scheme 5, (a)]. The catalytic mechanism was elucidated in detail by DFT^57^ and QM/MM calculations,^58^ kinetic isotope effects,^59^ as well as mechanism‐based inhibitor studies^60^ and was shown to involve an intermolecular 1,3‐dipolar cycloaddition step. The polarized C=C bond of the acrylic acid substrate acts as a dipolarophile, which undergoes a [3+2] cycloaddition with the azomethine ylide dipole in prFMN^iminium^, forming a five‐membered, pyrrolidine‐type substrate‐cofactor intermediate [Scheme 5, (a), (i)]. Decarboxylation is accompanied by fragmentation [Scheme 5, (a), (ii)], which allows for delocalization of the negative charge within the aromatic pyrimidine and carbonyl groups of the cofactor. This negative charge is utilized to reform the five‐membered product–cofactor adduct with concomitant protonation at C_α_ of the intermediate [Scheme 5, (a), (iii)] by a catalytic acid within the conserved Glu‐Arg‐Glu motif (Glu282 in *An*Fdc1). Finally, retro [3+2] dipolar cycloaddition as the rate‐limiting step^57^, ^59^ liberates the decarboxylated alkene [Scheme 5(a), (iv)]. Overall, the prFMN cofactor allows for delocalization of the accumulating negative charge to enable the stabilization of anionic intermediates during the decarboxylation process.

**Figure 4:**
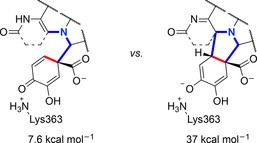
DFT‐calculated energies of the mono‐covalently *versus* di‐covalently bound substrate–cofactor intermediate compared to the **E:S** complex.^56^

A distinct mechanism was proposed for the (de)carboxylation of protocatechuic acid‐type substrates catalyzed by prFMN‐dependent 3,4‐dihydroxybenzoic acid decarboxylases (AroY) [Scheme 5, (c)]. Based on DFT calculations, the 1,3‐dipolar cycloaddition mechanism was ruled out due to prohibitively high energy barriers arising from the generation of a highly strained intermediate (Figure 4). Instead, the electrophilic character of the iminium ion of prFMN^iminium^ enables reversible decarboxylation *via* a mono‐covalently bound quinoid–cofactor intermediate with feasible energy barriers. Initiated by deprotonation of the phenolic OH group by a lysine residue, an electron flow across the protocatechuic acid substrate facilitates nucleophilic attack of C‐1′ onto the iminium ion in prFMN^iminium^. Formation of the mono‐covalently bound quinoid substrate–cofactor intermediate is hence accompanied by breaking of the aromaticity [Scheme 5, (c), (i)] and a subsequent (de)protonation/re‐aromatization sequence [Scheme 5, (c), (ii)–(iv)] furnishes the decarboxylated catechol. The residues Glu‐289 and Lys‐363 are within H‐bond distance (∼4 Å) and are assumed to be responsible for the acid–base catalytic steps.^56^

#### 3.3.2 Substrate Scope of (Putatively) prFMN‐Dependent Decarboxylases

**Figure 5:**
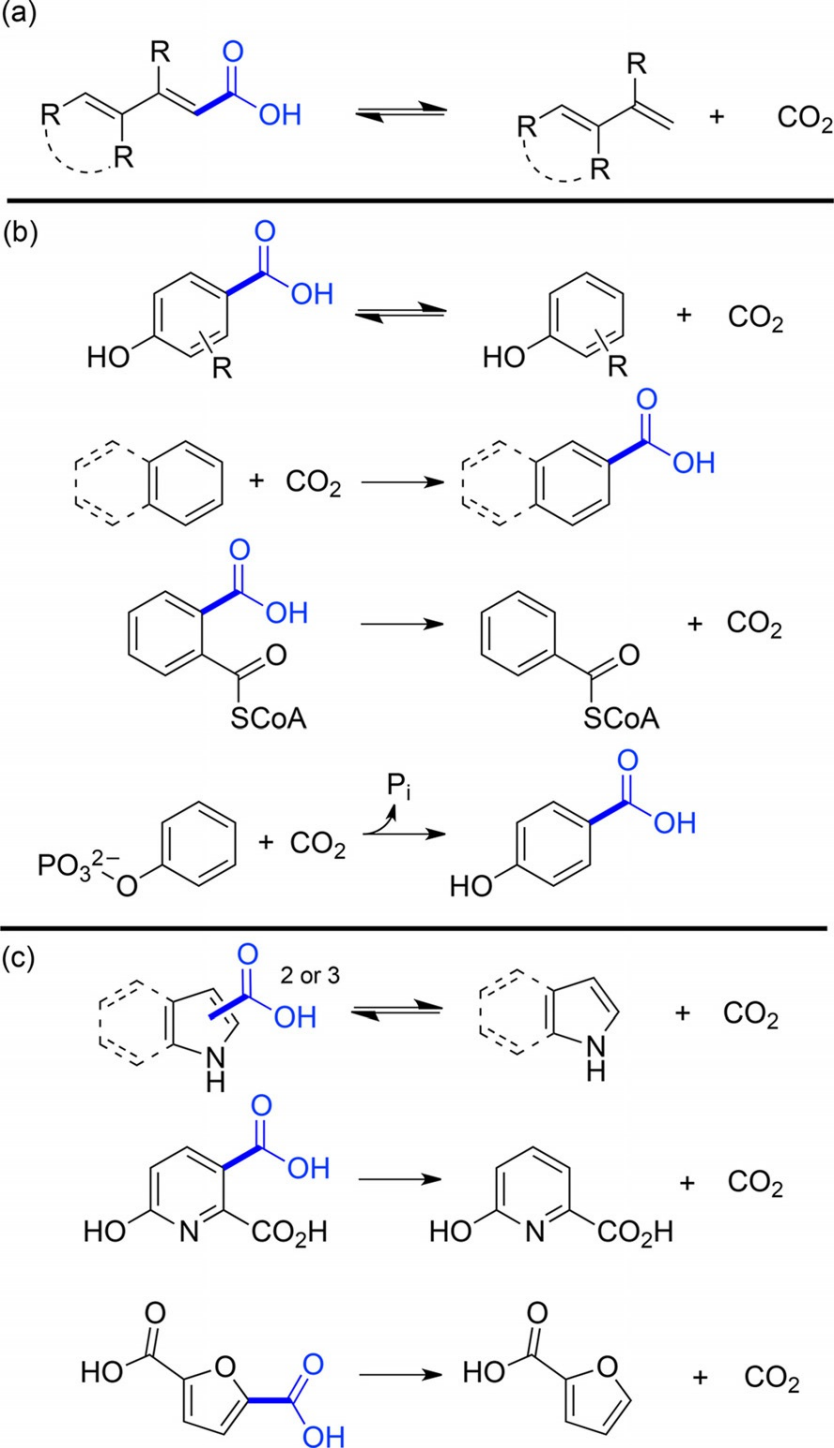
Types of substrates of (putatively) prFMN‐dependent (de)carboxylation reactions.

**Figure 6:**
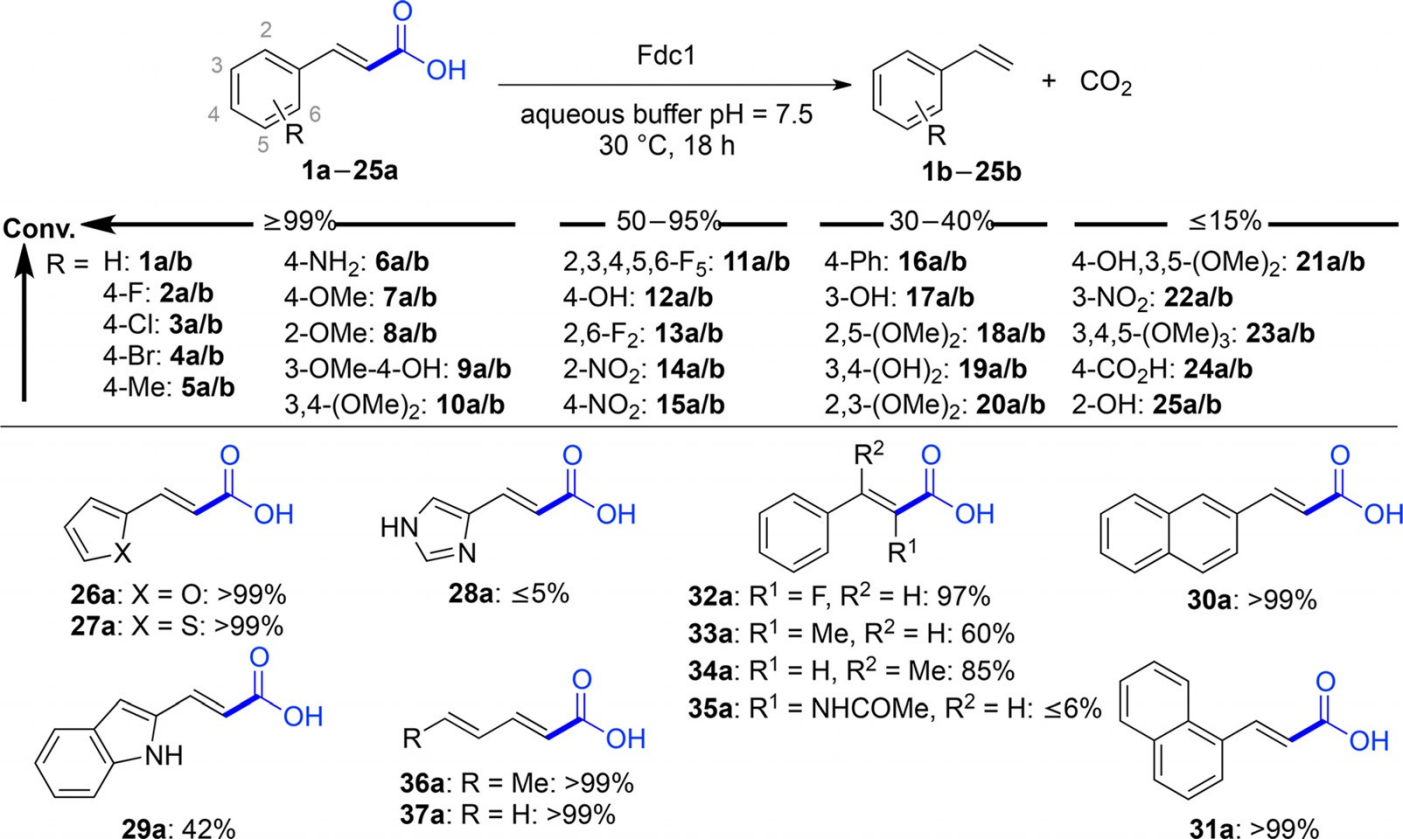
Substrate scope of *An*Fdc, *Sc*Fdc and *Cd*Fdc (applied as purified enzyme or lyophilized *E. coli* whole‐cell preparation containing the heterologously expressed decarboxylase).

**Figure 7:**
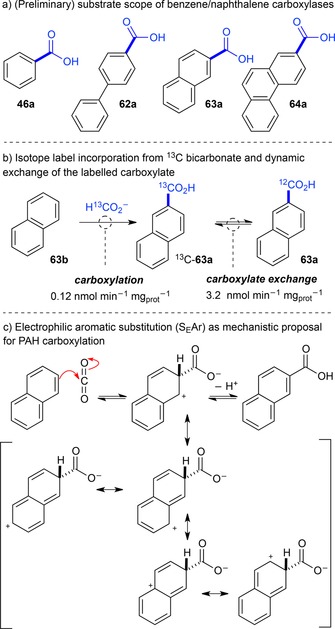
Enzymatic carboxylation of PAHs.

**Figure 8:**
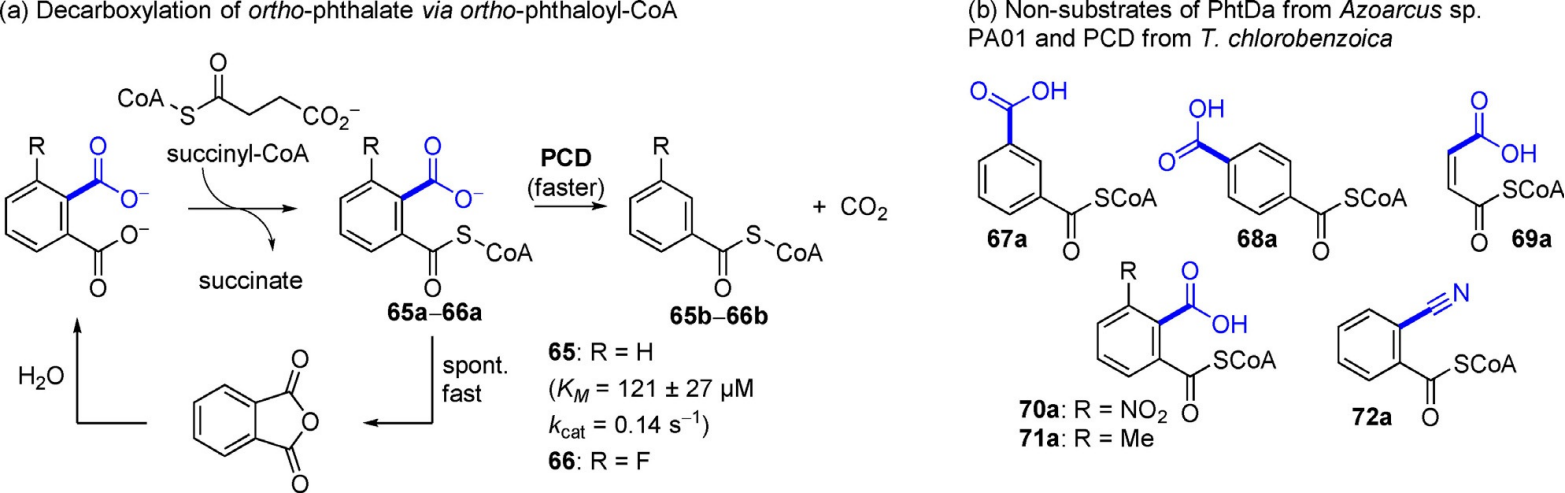
Phthalate decarboxylases.

**Figure 9:**
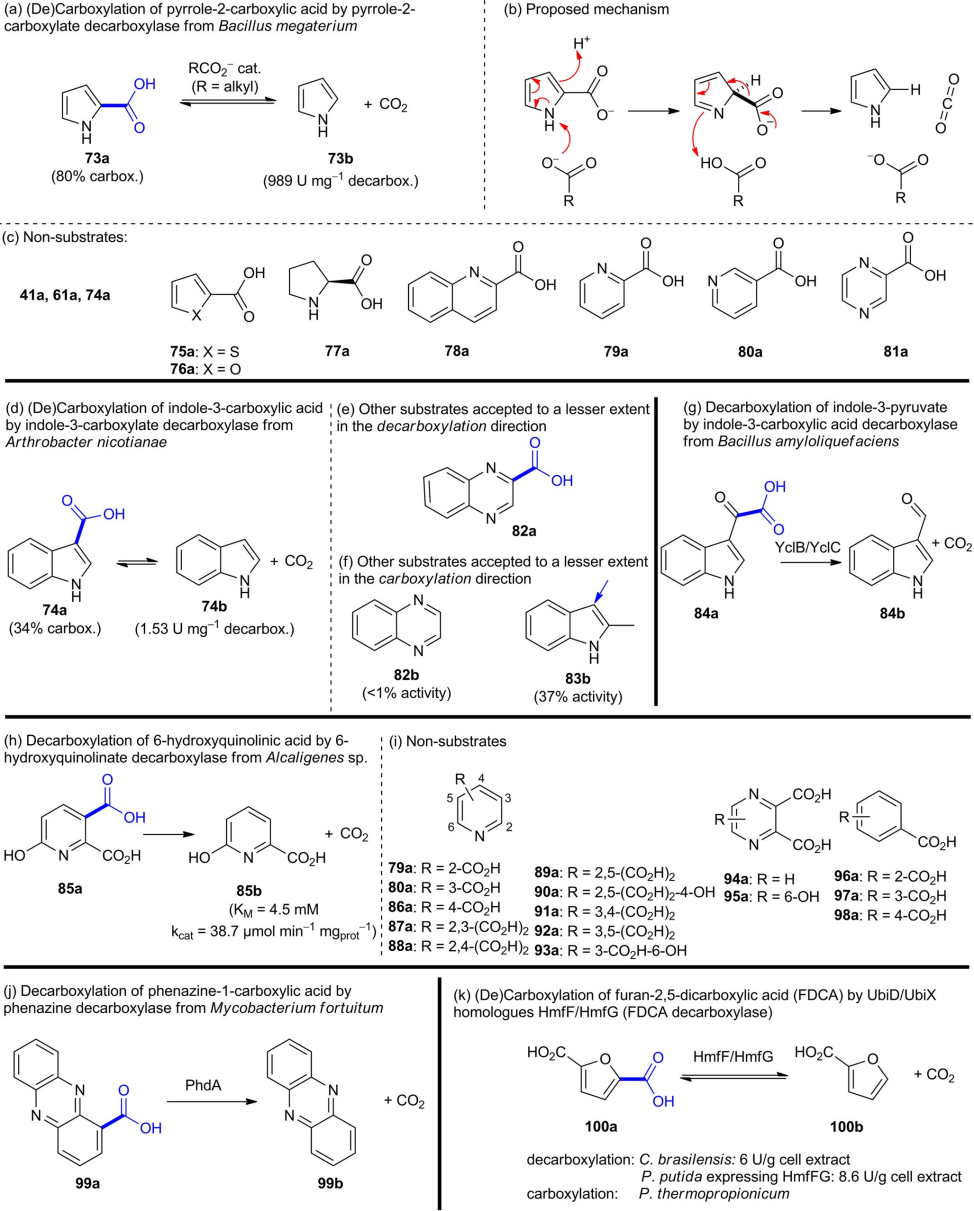
Substrate portfolio of heteroarene carboxylate decarboxylases.

Although only a few UbiD members (AroY, Fdc, phthtalate decarboxylase) have been proven to depend on prFMN until now, sequence comparison and gene organization strongly suggest that this cofactor is also involved in the (de)carboxylation of other (hetero)arene carboxylate substrates.22a Figure 1 (iii) gives a current overview of enzymatic (de)carboxylation reactions catalyzed by (putatively) prFMN‐dependent UbiD decarboxylases which encompass the following types of substrates (Figure 5): (a) reversible (de)carboxylation of non‐aromatic α,β‐unsaturated (acrylic) acid derivatives (Figure 6), (b) activated and non‐activated aromatic substrates, such as reversible *para*‐(de)carboxylation of catechol (Table 1) and 4‐hydroxybenzoic acid derivatives (4‐HBAs, Table 2), decarboxylation of phthaloyl‐CoA (Figure 8), carboxylation of phenyl phosphate going hand in hand with phosphate ester cleavage and carboxylation of polycyclic, aromatic hydrocarbons representing CO_2_‐fixation under anaerobic conditions (Figure 7), (c) reversible (de)carboxylation of heterocyclic substrates, such as pyrrole‐2‐carboxylic and indole‐3‐carboxylic acid, and decarboxylation of 6‐hydroxyquinolinic and 2,5‐furandicarboxylic acid (Figure 9).

**α**,**β*‐Unsaturated (acrylic) acids***: While Fdc1 is responsible for the decarboxylation of cinnamic, ferulic and 2,4‐hexanedienoic (sorbic) acid *in vivo*,^61^ a broad spectrum of non‐natural substituted acrylic acids was readily decarboxylated by *ubiX*‐coexpressed enzymes from *Candida dubliniensis* (*Cd*Fdc), *Aspergillus niger* (*An*Fdc) and *Saccharomyces cerevisiae* (*Sc*Fdc) in purified form or *E. coli* whole‐cell preparations. Depending on the structure and electronic nature of the substrates, conversions ranged from excellent (≥99%), good (50–95%) to moderate (30–40%) and poor (≤15%) (Figure 6).^62^ Numerous cinnamic acid derivatives were well accepted by Fdcs tolerating a variety of substitutions at the aromatic core and also to some extent at the α,β‐C=C bond involved in the [3+2] cycloaddition. The electronic nature of the *para*‐substituents is highly flexible (conversion ≥99%), including weak (4‐Me) and strong (4‐NH_2_, 4‐OMe, 4‐OH) e^−^‐donating as well as weakly e^−^‐withdrawing (4‐halogen) groups. Fdcs also accept substrates lacking *para*‐substitutuents, which is in stark contrast to PADs, which need an activating *p*‐OH group. Only strong e^−^‐withdrawing (4‐NO_2_, 4‐CO_2_H) and more bulky *para*‐substituents (4‐Ph) hampered the decarboxylation activity to some extent. Substitution in *meta*‐ and *ortho*‐positions of the aromatic ring is less flexible compared to the *para*‐position. In the *meta*‐position weak e^−^‐donating groups (3‐OMe) are most favoured whereas strong e^−^‐donating substituents (3‐OH) lead to lower conversions. The *ortho*‐position is rather restricted to small and less polar (F, Me) groups leading to reduced decarboxylation in the case of larger (OMe) and more polar (NO_2_, OH) substituents. Furthermore, these enzymes also accepted heterocyclic analogues of cinnamic acid as substrates (Figure 6, **26a**–**29a**). Substitution at the α‐ or β‐carbon atom is well accepted by the enzymes, a further difference to PADs, as long as they are not too bulky (Figure 6, **32a**–**35a**). Non‐aromatic conjugated 2,4‐dienoic acids (Figure 6, **36a**, **37a**) were well accepted, the latter being a valuable extension of the substrate panoply for biocatalytic decarboxylations and allowing access to 1,3‐dienes from the corresponding fatty acids. In addition, kinetic parameters of *An*Fdc^50^ and *Sc*Fdc^59^ with selected cinnamic acid substrates were reported.

Overall, the minimum structural substrate requirements are marked by the obligatory conjugation of the *E*‐configured α,β‐C=C bond involved in the 1,3‐dipolar cycloaddition to (at least) one other C=C bond, which may be part of a larger (aromatic) π‐system.

Attempts to carboxylate alkenes matching the minimal substrate requirements (using bicarbonate or CO_2_ as co‐substrate) were unsuccessful.^62^

**Scheme 6:**
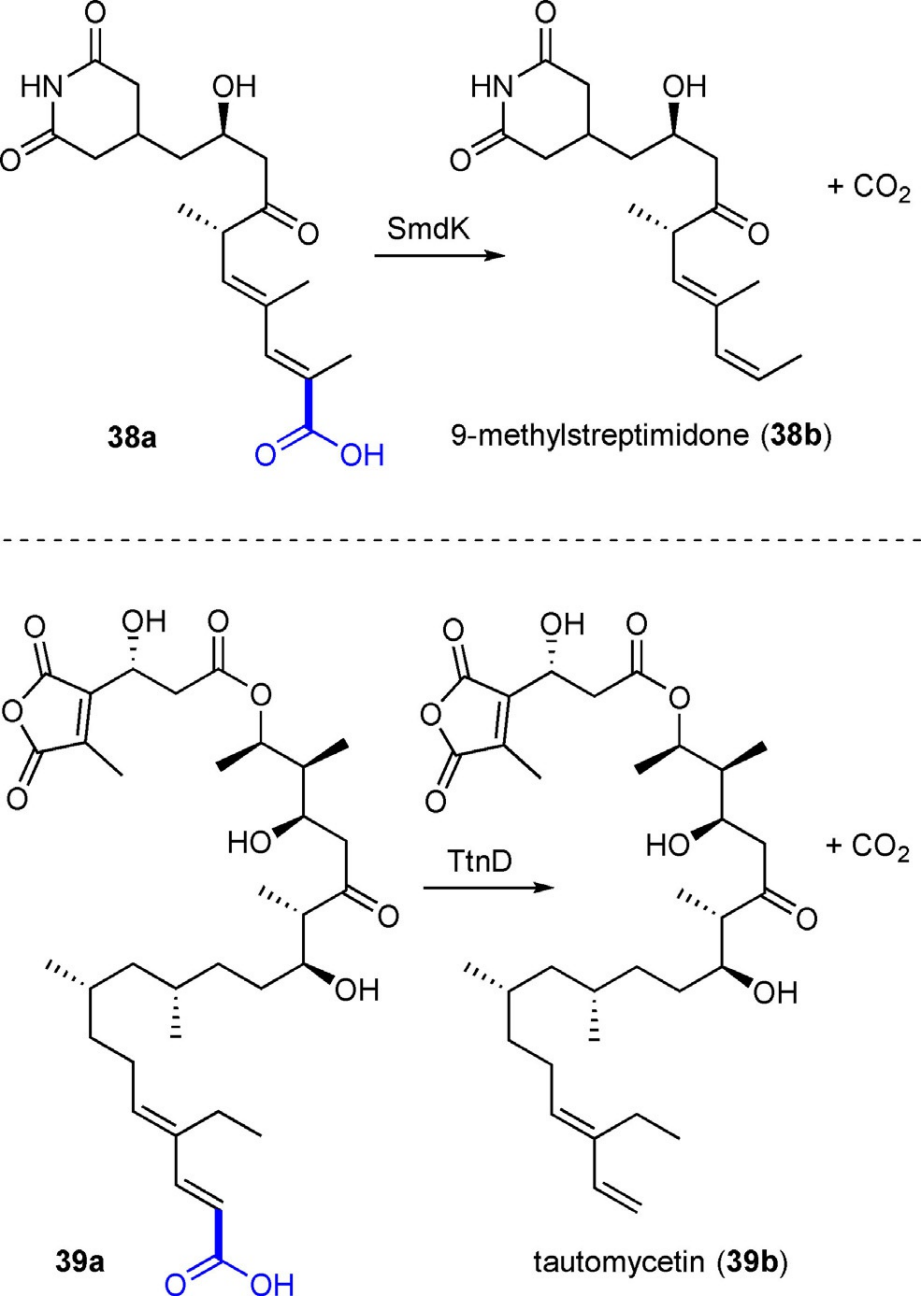
SmdK and TtnD‐catalyzed decarboxylation in the biosynthesis of polyketides.

Two related decarboxylases, SmdK^63^ (34% identity to *Sc*Fdc) involved in the biosynthesis of the polyketide 9‐methylstreptimidone (**38b**, Scheme 6) from *Streptomyces himastatinicus* and TtnD^64^ from *Streptomyces griseochromogenes* involved in the biosynthesis of the polyketide tautomycetin (**39b**, Scheme 6) act on large polyfunctionalized 2,4‐diunsaturated carboxylic acids illustrating the versatility of this enzyme class.

***Protocatechuic acid/catechol‐type substrates and 4‐hydroxybenzoic acids***: In contrast to the synthetically very versatile *ortho*‐(de)carboxylation by *o*‐BDCs (see above), studies on the substrate profile of the regio‐complementary *para*‐(de)carboxylation of phenolic substrates are rare.^53^, ^56^, ^65^ Two protocatechuic acid decarboxylase homologues, 3,4‐dihydroxybenzoic acid decarboxylases (AroY) from *Enterobacter cloacae* (*Ec*AroY) and *Klebsiella pneumoniae* (*Kp*AroY), which show high similarity in structure and sequence (89% sequence identity), were explored in the reversible (de)carboxylation of catechol‐type substrates. A set of substituted catechol and protocatechuic acid derivatives was applied to evaluate the substrate tolerance of these enzymes employed as lyophilized *E. coli* whole‐cell preparations harbouring the heterologously expressed decarboxylase and endogeneous UbiX for providing prFMN (Table 1).

**Table 1:** Substrate scope of 3,4‐dihydroxybenzoic acid decarboxylase from *E. cloacae* (*Ec*AroY).^[a]^ 
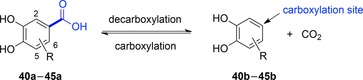

Entry	Decarboxylation			Entry	Carboxylation	
	Substrate	Conversion [%]			Substrate	Conversion [%]
1	**40a** R=H	>99		4	**40b** R=H	16/6^[b]^
2	**41a** R=5‐OH	>99		5	**41b** R=5‐OH	15
3	**42a** R=2‐OH	<1		6	**43b** R=5‐F	13
	–			7	**44b** R=5‐OMe	2
	–			8	**45b** R=5‐Me	9

^[a]^ *Ec*AroY substrate screening with lyophilized *E. coli* whole cells containing the heterologously expressed decarboxylase using potassium bicarbonate (3 M) as CO_2_ source. ^[b]^ Pressurized CO_2_ (30 bar) was used as CO_2_ source in the carboxylation mode.

The assumed natural substrate (**40a**, protocatechuic acid) and the *meta*‐substituted (5‐OH, **41a**, gallic acid) derivative were quantitatively decarboxylated, whereas no reaction was observed with isogallic acid (**42a**). Interestingly, also the reverse, thermodynamically disfavoured carboxylation reaction of several catechol derivatives was observed in the presence of bicarbonate (3 M) or pressurized CO_2_ (30 bar) as CO_2_ source (Table 1, entries 4–8). Overall, substrate profile studies illustrated that the *meta*‐position is most flexible and tolerates e^−^‐withdrawing and ‐donating substituents (5‐OH, ‐F, ‐OMe, ‐Me, **41b**–**45b**) whereas substitution in the *ortho*‐position (2‐OH, **42a**) was not accepted for steric reasons. The catechol motif (two hydroxy groups) seems to be crucial, since substituted phenols were not accepted as substrates.

Besides the biocatalytically characterized prFMN‐dependent AroY enzymes, related UbiD‐like (and hence putatively prFMN dependent) *para*‐carboxylases from various microbial sources can be found in the literature. Table 2 gives an overview of (partially characterized) *para*‐carboxylases and their substrate and non‐substrate scope. The preference for a certain substrate allows classification into 4‐hydroxybenzoate, protocatechuate (including AroY), gallate and vanillate decarboxylases. However, the oxygen sensitivity of some of these proteins severely impedes their application in biotransformations.

**Table 2:** Substrate scope of 4‐hydroxybenzoate decarboxylases (EC 4.1.1.61), protocatechuate decarboxylases (EC 4.1.1.63), gallate decarboxylases (EC 4.1.1.59) and vanillate decarboxylases (no EC number assigned). Substrates with highest activities are marked with an asterisk (*). 
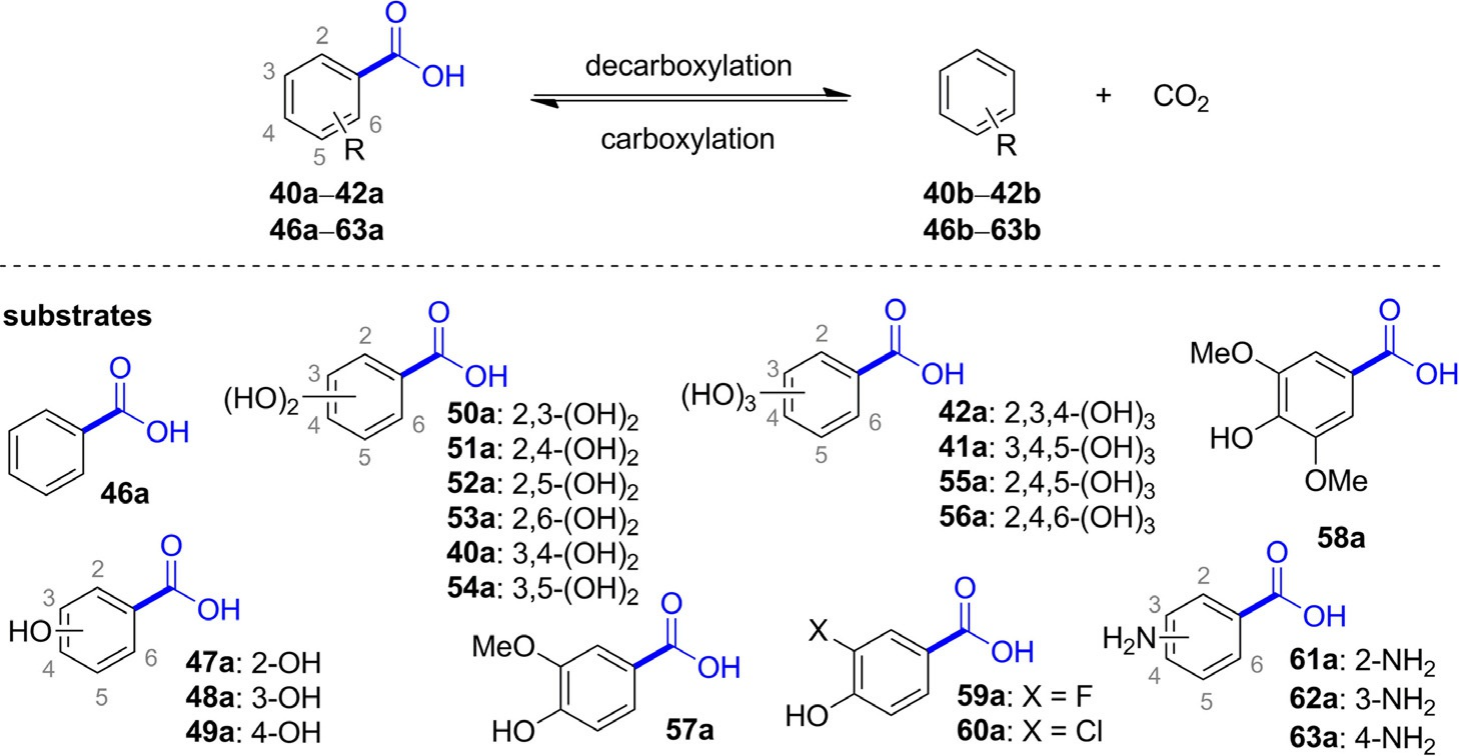

Organism (enzyme name)	Accepted substrates	Tested non‐ substrates	Ref.
***4‐Hydroxybenzoate decarboxylases***			
*Sedimentibacter hydroxybenzoicus* JW/Z1 (basonym *Clostridium hydroxybenzoicum*)^[b,c,d]^	**49a**,*^[e]^ **40a**,^[e]^ **57a**, **59a**, **60a**	**47a**, **48a**, **50a**, **51a**, **52a**, **42a**, **41a**	66
*Cryptoanaerobacter phenolicus* ^[c,d]^	**49a**	**48a**	67
*Enterobacter cloacae* P240 (4‐HBD)^[b]^	**49a**,*^[e]^ **40a**	**46a**, **47a**, **48a**, **53a**, **56a**, **41a**, **57a**, **63a**	68
*B. subtilis* (*Bs*dBCD)^[a]^	**49a**,*^[e]^ **57a** ^[e]^	–	65, 69
*E. coli* O157:H7 (*Ec*dCD)^[a]^	**49a**,*^[e]^ **57a** ^[e]^	–	65, 70
*Salmonella typhimurium* (*St*dBCD)^[a]^	**49a**,*^[e]^ **57a** ^[e]^	–	65
*Chlamydia pneumoniae* AR39^[b,d]^	**49a** ^[e]^	**51a**	71
*Klebsiella aerogenes* (*Klebsiella pneumoniae*) (*Kp*BCD)^[a,c]^	**49a**,*^[e]^ **52a**, **40a**, **41a**, **57a** ^[e]^	**46a**, **47a**, **48a**, **50a**, **51a**, **53a**, **54a**, **42a**, **61a**, **62a**, **63a**, **56a**	65, 72
*Desulfovibrio* sp./*Methanospirillium hungatei* consortium^[b,d]^ (two decarboxylases, one carboxylase)	**49a**,* **41a**, **59a**, **60a**	–	73
*Clostridium thermoaceticum* (basonym *Moorella thermoacetica*)^[b,c,d]^	**49a**,* **40a**, **57a**, **60a**, **59a**	**46a**, **47a**, **48a**, **50a**, **51a**, **52a**, **53a**, **54a**, **41a**, **58a**	74

***Protocatechuate decarboxylases***			
*Enterobacter cloacae* P240 (*Ec*AroY)^[b]^	**40a** ^[e]^	–	75
*Sedimentibacter hydroxybenzoicus* JW/Z1 (basonym *Clostridium hydroxybenzoicum*) (3,4‐DHBD)^[b,c,d]^	**40a** ^[e]^	**47a**, **48a**, **49a**, **50a**, **51a**, **52a**, **42a**, **41a**, **57a**, **59a**	66a, 76

***Gallate decarboxylases***			
*Citrobacter* sp.^[c]^	**40a**, **41a**,* **54a**, **48a**, **50a**	**46a**, **47a**, **51a**, **52a**, **53a**, **41a**, **61a**, **62a**	77
*Arxula adeninivorans* (*Ag*dc1p)^[b]^	**40a**, 41a*	**48a**–**52a**	78
*Lactobacillus plantarum Lp*dC^[a]^ (UbiX‐like *Lp*dB required for activity)	**40a**, 41a*	–	79
*Pantoea agglomerans* T71^[b,d]^	**41a** ^[f]^	**46a**, **40a**–**42a**, **46a**–**57a**, **62a**, **63a**	80

***Vanillate decarboxylase***			
*Streptomyces* sp. D7 (VcdCD)^[a,b]^	**57a** ^[e]^	**49a**, **40a**, **41a**, **58a**	65, 81

^[a]^ Recombinant decarboxylase expressed in *E. coli*. ^[b]^ (Partially) purified from wild‐type strain. ^[c]^ (Induced) resting cells (*in vivo* decarboxylation). ^[d]^ Oxygen‐sensitive protein and/or anaerobic strain. ^[e]^ Reverse carboxylation of the respective phenol in the presence of excess bicarbonate was demonstrated. ^[f]^ Carboxylation was tested, but no product acid was detected.

Recent discoveries in the organization of ubiD/ubiX genes and other associated genes encoding for proteins of yet unknown function,22a, 65 as well as the knowledge of *in vitro* activation with the catalytically active cofactor outlined above opens new possibilities for prFMN‐dependent phenol *para*‐carboxylases for biocatalytic purposes.

***Polycyclic aromatic hydrocarbons (PAHs)***: The degradation of polycyclic aromatic hydrocarbons (PAHs) in polluted soil and aquifers under anoxic conditions proceeds *via* enzymatic carboxylation as one of the initial steps towards mineralization of these environmental hazards. Meckenstock et al. provide a comprehensive overview of the recent developments in this field, with a special focus on microbiology, ecology, and biochemistry.^82^ Although research is still in its infancy, these enzymes pose highly interesting biocatalysts given their potential ability to directly functionalize chemically non‐activated aromatics under mild, anoxic conditions to yield the corresponding carboxylic acids without the need for ATP or other expensive co‐substrates. Carboxylated metabolites of benzene,^83^ biphenyl,^84^ naphthalene^85^ and phenanthrene^86^ were detected in supernatants of anaerobic sulfate‐reducing enrichment cultures [Figure 7, (a)]. In a more detailed study with crude cell extracts of culture N47, the carboxylation of naphthalene in the 2‐position was demonstrated to involve incorporation of ^13^C bicarbonate into the product. Dynamic enzyme‐catalyzed carboxylate ^13^C‐isotope label exchange suggests reversibility of the process [Figure 7, (b)].^87^ How exactly carboxylation of non‐activated aromatics lacking electron‐donating substituents is accomplished in the active site of these enzymes is a subject of ongoing research. A crude mechanistic proposal suggests nucleophilic attack at the 2‐position of the arene with CO_2_ as electrophile, while the resulting carbocation is delocalized in the (annulated) aromatic system (S_E_Ar) [Figure 7, (c)].^82^ In any case, the carboxylation of non‐activated aromatics is a ‘difficult’ reaction, as domonstrated by the very low reaction rates.^82^

Genetic studies of the benzene‐induced putative anaerobic benzene carboxylases reveal a cluster organization with two subunits (abcA and abcD) and a gene sequence similar to that of ATP‐dependent phenyl phosphate carboxylases from *A. aromaticum* of the UbiD family.^88^ In addition, genes similar to the ubiD/ubiX system were highly transcribed in benzene‐degrading nitrate‐reducing enrichment cultures,^89^ which suggests a possible role of prFMN in the carboxylation of PAHs.

***Phthaloyl‐CoA decarboxylases***: The anaerobic degradation of *ortho*‐phthalic acid formed from the corresponding esters originating out of plasticizers of domestic origin^90^ features yet another class of decarboxylases (Figure 8). Recent pioneering studies showed that cell extracts of *Azoarcus* strain PA01 (phtDa),^91^ *Azoarcus evansii* KB740, *Aromatoleum aromaticum* EbN1, and *Thauera chlorobenzoica* 3CB‐1 anaerobically grown on phthalate as sole carbon source are catalyzing the formation of benzoyl‐CoA with succinyl‐CoA as co‐substrate. Among the upregulated genes, homologues of *ubiD* and a succinyl‐CoA:*ortho*‐phthalate‐CoA transferase are involved in the anaerobic conversion of *ortho*‐phthalate to benzoyl‐CoA.^92^

In order to intercept the labile phthaloyl‐CoA intermediate, decarboxylation must proceed at a faster rate than the spontaneous formation of phthalic anhydride, which recycles to phthalate through hydrolysis. Whereas succinyl‐CoA:*ortho*‐phthalate‐CoA transferase is highly substrate specific, PCD accepted 3‐flouro‐*ortho*‐phthaloyl‐CoA as substrate with 16% activity of the native substrate. Since the reverse carboxylation of benzoyl‐CoA in the presence of bicarbonate (2 M) and isotope exchange between ^13^C‐bicarbonate and the substrate failed, decarboxylation appears to be irreversible.^93^ In any case, the requirement for CoA‐activated substrates excludes these (biochemically interesting) enzymes from practical application.

In order to explain the involvement of a CoA‐substrate conjugate, an activating role of the thioester linkage (in analogy to the 1,3‐dipolar cycloaddition in Fdc1) was proposed, where the C=C bond between the carboxylate moieties acts as dipolarophile, while the negative charge is delocalized across the cofactor's isoalloxazine ring.93a

***Heteroaromatic substrates***: The first example for a non‐oxidative, reversible decarboxylation of a heteroaromatic carboxylate is the conversion of pyrrole‐2‐carboxylic acid (**73a**) to pyrrole (**73b**) by pyrrole‐2‐carboxylate decarboxylase from *Bacillus megaterium* PYR2910 [Figure 9, (a)].^94^ The synthetic utility of the reverse carboxylation of pyrrole was demonstrated using excess bicarbonate (80% conversion, 52% isolated yield),^95^ pressurized CO_2_ in a flow‐setup (24±7 μmol/h space‐time yield),^96^ or supercritical CO_2_ (59% conversion).^97^ Since the activity depends on the presence of (small) organic acids, a mechanism including an external carboxylate acting as catalytic base was proposed [Figure 9, (b)]. However, other heterocyclic substrates fulfilling the structural requirements for this mechanism to happen (except **77**) were not converted [Figure 9, (c)].^94^

Indole‐3‐carboxylate decarboxylase from *Arthrobacter nicotianae* catalyzes the reversible decarboxylation of indole‐3‐carboxylic acid [Figure 9, (d)–(g)]. Resting cells are able to catalyze the reverse carboxylation of indole **74b** and 2‐methylindole **83b** at the expense of bicarbonate yielding the corresponding heteroaromatic carboxylic acids (34% conversion of **74b**). Only trace activity with quinoxaline‐2‐carboxylic acid (**82a**) and quinoxaline (**82b**) in the carboxylation and decarboxylation direction was detected.^98^ Primary sequence data are still elusive for both pyrrole‐2‐carboxylate and indole‐3‐carboxylate decarboxylases, therefore no conclusions on their role within the UbiD superfamily can be drawn.

Heteroarene decarboxylases with sequences related to other UbiD members22a, 99 [see Figure 1, (iii)], and hence putative dependency on prFMN, have been discovered but the mechanistic role of this cofactor in the decarboxylation of heterocyclic substrates is unknown so far.

An UbiD‐like decarboxylase, YclC from *Bacillus amyloliquefaciens*, is involved in the decarboxylation of indole‐3‐pyruvate in the biosynthesis of the phytohormone indole‐3‐acetic acid [Figure 9, (g)].^100^

Another enzyme purified from *Alcaligenes* sp. strain UK21 is involved in the highly substrate specific non‐oxidative decarboxylation of 6‐hydroxyquinolinate (**85a**) to 6‐hydroxypicolinic acid (**85b**) [Figure 9, (h)–(i)]. The decarboxylase shows some similarity to phthalate decarboxylases of the UbiD family, although no requirement for cofactors was reported. Attempts to run the process in the reverse carboxylation direction were unsuccessful.^101^

Recently, also the decarboxylation of phenazine‐1‐carboxylic acid (**99a**) by the UbiD‐like enzyme PhdA from *Mycobacterium fortuitum* associated to the UbiX homologue PhdB was reported [Figure 9, (j)].^102^

The decarboxylation of furan‐2,5‐dicarboxylic acid (**100a**) in the furoic alcohol‐metabolizing strain *Cupriavidus brasilensis* HMF14 is associated with the enzyme couple HmfF and HmfG [Figure 9, (k)]. Cell extracts of *C. brasilensis* and *P. putida* heterologously expressing HmfF/G catalyzed the single decarboxylation of **100a** to 2‐furoic acid (**100b**). Expressing either the isolated UbiD‐homologue HmfF or the UbiX‐homologue HmfG in *P. putida* resulted in no or significantly reduced decarboxylation activity,^103^ underpinning a putative requirement of UbiX‐derived prFMN. In the presence of elevated concentrations of CO_2_ [1 M bicarbonate and/or pressurized CO_2_ (32 bar)] the reversed carboxylation reaction of 2‐furoic acid (**100b**) to **100a** was observed employing FDCA (2,5‐furandicarboxylic acid) decarboxylase (HmfF/HmfG) from *Pelotomaculum thermopropionicum* albeit with low yield.^104^

In retrospect, little is known about biocatalytic heteroarene (de)carboxylation as of yet, but with the advent of prFMN‐dependent catalysis and the knowledge available for providing active reconstituted enzymes, the conversion of these substrates by UbiD‐family members poses an interesting research aim.

## 4 Conclusion

Overall, enzyme‐catalyzed (de)carboxylation concepts have been established as suitable synthetic tools, using carbon dioxide as C_1_‐building block for the production of valuable chemicals. The biocarboxylation of electron‐rich (hetero)arenes proceeds under mild reaction conditions in a highly regioselective fashion. As such, these methods offer particular benefits over chemical methods like the Kolbe–Schmitt reaction, which often require high pressure and temperature and suffer from incomplete regioselectivities.

Metal‐dependent *ortho*‐benzoic acid decarboxylases and cofactor‐independent phenolic acid decarboxylases are remarkably stable under biotransformation conditions, and have been exploited for the regioselective carboxylation of a range of electronically and structurally diverse phenolic and coumaric acid derivatives, respectively. Despite that, process engineering needs to be further developed to drive the equilibrium in the thermodynamically disfavoured carboxylation direction in order to facilitate large‐scale applications.

Ever more prenylated FMN‐dependent decarboxylases of the widespread UbiD family are being discovered, which catalyze highly interesting preparative reactions. Examples include the (de)carboxylation of (hetero)aromatic substrates and extension of the biocatalytic toolbox towards the decarboxylation of cinnamic and 2,4‐hexadienoic acid derivatives. Nonetheless, due to the recent discovery of the modified FMN cofactor the investigation of the chemistry implied by the prFMN structure is a subject of ongoing research. Hence, at present several limitations for prFMN‐dependent decarboxylases need to be addressed in order for this highly interesting enzyme class to become a widely applicable biocatalytic tool for organic synthesis. These limitations include, but are not limited to, the light sensitivity of prFMN^iminium^, the O_2_ sensitivity of some UbiD family members, co‐expression of the prenyl transferase UbiX, as well as efficient *in vitro* reconstitution of *apo*‐decarboxylases as well as the role of other allosteric factors during catalysis.

## Biographical Information

*Stefan E. Payer*, born 1989 in Leoben, Austria, obtained his master's degree under the supervision of Prof. Wolfgang Kroutil at the University of Graz working on the chemoenzymatic synthesis of pyrrolizidine alkaloids and the development of “smart co‐substrates” for biocatalytic transamination reactions. During his Ph.D. work under the supervision of Prof. Kurt Faber and Silvia M. Glueck, he turned to investigate various carboxylase biocatalysts and their use as stereoselective vinylphenol hydratases as well as the prFMN‐dependent *para*‐carboxylation of catechols. Currently, he is an FWF‐Schrödinger postdoctoral fellow at the University of California, Berkeley, CA, USA.

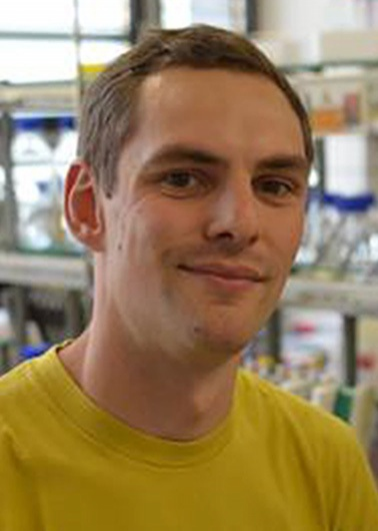

## Biographical Information

*Kurt Faber* completed his studies in chemistry with a Ph.D. in 1982 at the University of Graz. After a post‐doctoral fellowship (1982–1983) in St. John's (Canada) he moved to the University of Technology in Graz, where he became associate professor in 1997. The following year he was appointed full professor at the University of Graz, where he headed a research group devoted to the use of biocatalysts for the transformation of non‐natural compounds until his retirement in 2017. He was a visiting scientist at the University of Tokyo (1987–1988), Exeter University (1990), the University of Trondheim (1994), Stockholm University (2001), the University of Minnesota (2005), the ESPCI in Paris (2010) and the University of Pavia (2019). As senior scientist he continues to promote the development of biotransformations for synthetic organic chemistry.

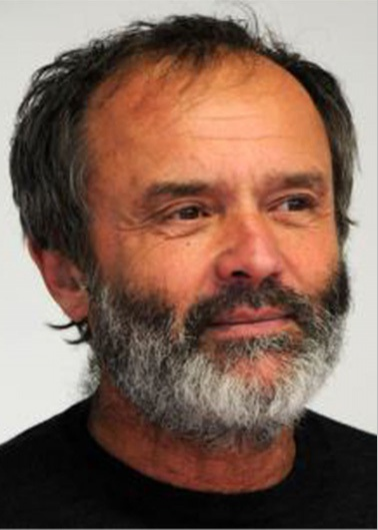

## Biographical Information

*Silvia M. Glueck*, born 1973, studied chemistry at the University of Graz, Austria, where she received her Ph.D. degree under the supervision of Prof. Kurt Faber in 2004. After a postdoctoral stay at the Universities of Edinburgh and Manchester (2005–2007) with Prof. Nicholas J. Turner, she returned to Graz as scientist and was later appointed as senior scientist within the Austrian Centre of Industrial Biotechnology (acib) and the University of Graz. She currently holds a position as university assistant in the Department of Chemistry at the University of Graz. Her research interests focus on biocatalytic synthesis as alternative to chemical systems, in particular, (de)carboxylation and asymmetric hydration reactions.